# MITF is a novel transcriptional regulator of the calcium sensor STIM1: Significance in physiological melanogenesis

**DOI:** 10.1016/j.jbc.2022.102681

**Published:** 2022-11-07

**Authors:** Jyoti Tanwar, Akshay Sharma, Suman Saurav, Shyamveer, Nidhi Jatana, Rajender K. Motiani

**Affiliations:** 1 Laboratory of Calciomics and Systemic Pathophysiology (LCSP), Regional Centre for Biotechnology (RCB), Faridabad, Delhi-NCR, India; 2 CSIR-Institute of Genomics and Integrative Biology (IGIB), New Delhi, India; 3 Academy of Scientific and Innovative Research (AcSIR), Ghaziabad, India; 4 Indian Biological Data Centre (IBDC), Regional Centre for Biotechnology (RCB), Faridabad, Delhi-NCR, India

**Keywords:** STIM1, MITF, calcium signaling, store operated calcium entry, melanogenesis, αMSH, α-melanocyte stimulating hormone, cAMP, cyclic-AMP, ChIP, chromatin immunoprecipitation, CREB, cAMP response element binding protein, DCT, Dopachrome Tautomerase, EPD, Eukaryotic Proteome Database, ER, endoplasmic reticulum, FBS, fetal bovine serum, HD, high density, LD, low density, MITF, microphthalmia-associated transcription factor, NFDM, non-fat dry milk, NT, nontargeting, PWM, position weight matrix, SOCE, store-operated Ca2+ entry, STIM1, Stromal Interaction Molecule1, Tg, thapsigargin, TTBS, tris-buffered saline containing 0.1% Tween 20

## Abstract

Stromal Interaction Molecule1 (STIM1) is an endoplasmic reticulum membrane-localized calcium (Ca^2+^) sensor that plays a critical role in the store-operated Ca^2+^ entry (SOCE) pathway. STIM1 regulates a variety of physiological processes and contributes to a plethora of pathophysiological conditions. Several disease states and enhanced biological phenomena are associated with increased STIM1 levels and activity. However, molecular mechanisms driving STIM1 expression remain largely unappreciated. We recently reported that STIM1 expression augments during pigmentation. Nonetheless, the molecular choreography regulating STIM1 expression in melanocytes is completely unexplored. Here, we characterized the molecular events that regulate STIM1 expression during pigmentation. We demonstrate that physiological melanogenic stimuli α-melanocyte stimulating hormone (αMSH) increases STIM1 mRNA and protein levels. Further, αMSH stimulates STIM1 promoter-driven luciferase activity, thereby suggesting transcriptional upregulation of STIM1. We show that downstream of αMSH, microphthalmia-associated transcription factor (MITF) drives STIM1 expression. By performing knockdown and overexpression studies, we corroborated that MITF regulates STIM1 expression and SOCE. Next, we conducted extensive bioinformatics analysis and identified MITF-binding sites on the STIM1 promoter. We validated significance of the MITF-binding sites in controlling STIM1 expression by performing ChIP and luciferase assays with truncated STIM1 promoters. Moreover, we confirmed MITF’s role in regulating STIM1 expression and SOCE in primary human melanocytes. Importantly, analysis of publicly available datasets substantiates a positive correlation between STIM1 and MITF expression in sun-exposed tanned human skin, thereby highlighting physiological relevance of this regulation. Taken together, we have identified a novel physiologically relevant molecular pathway that transcriptionally enhances STIM1 expression.

Stromal Interaction Molecule1 (STIM1) is a single transmembrane protein localized on endoplasmic reticulum (ER) (1). It is an ER calcium (Ca^2+^) sensor, which binds to Ca^2+^ through its EF hand domains (1). Upon decrease in ER Ca^2+^ levels, STIM1 oligomerizes and activates plasma membrane–resident Orai channels (Orai1/2/3) (1). The activation of Orai channels results in intracellular Ca^2+^ entry. Since ER Ca^2+^ stores depletion results in this Ca^2+^ influx, it is called as Store Operated Ca^2+^ entry (SOCE) (2). STIM1 and SOCE are widely studied in a variety of cellular systems wherein STIM1 plays a critical role in regulating cell physiology and human health (3). STIM1 is involved in various diseases associated with dysregulation of SOCE including vascular disorders, neurodegenerative conditions, severe combined immunodeficiency syndrome, skeletal muscle disorders, and cancers (4, 5, 6, 7, 8). STIM1 is overexpressed in a number of physiological phenomena including pigmentation (9). Mutations in STIM1 lead to various diseases such as severe combined immunodeficiency syndrome, autoimmunity, muscular hypotonia, tubular aggregate myopathy, Stormorken syndrome, and ectodermal dysplasia among others (10). STIM1 levels are significantly modulated in various acute and chronic neurodegenerative diseases such as ischemic brain injury and Alzheimer’s disease (11). Further, STIM1 expression is augmented in a plethora of pathological conditions including vascular disease (6, 12), asthma (13), and several types of cancer (4, 7, 14). Taken together, elevated STIM1 levels are associated with certain physiological phenomena as well as lead to a variety of pathological conditions. However, molecular mechanisms regulating STIM1 expression remain poorly understood.

Pigmentation is a complex physiological phenomenon that protects human skin from UV radiation–induced damage. This protection is mediated *via* a critical photo-protective factor known as melanin (15). Melanin is synthesized in melanosomes (dedicated organelle for melanogenesis), which are lysosome-related organelles within specialized cells called melanocytes (16, 17). The produced melanin is then transferred to neighboring keratinocytes, which provides protection to the skin from UV-induced cellular damage (18). α-melanocyte stimulating hormone (αMSH) is a key physiological inducer of melanogenesis in humans (19, 20). It is secreted downstream of UV exposure from keratinocytes and acts *via* binding to the G protein–coupled receptor melanocortin 1 receptor on melanocytes. This binding subsequently leads to induction of melanogenesis by activating cyclic-AMP (cAMP)-PKA-cAMP response element binding protein (CREB)-microphthalmia associated transcription factor (MITF) signaling axis (21). MITF is the master transcription factor that regulates both pigmentation and melanocyte proliferation (22, 23).

Our recent work implicated a critical role of STIM1 in pigmentation (9). Interestingly, we reported that the expression of STIM1 is higher in pigmented B16 cells (in B16 low density (LD) pigmentation model) than nonpigmented B16 cells (9). This data suggests that STIM1 levels augment as cells undergo pigmentation. But the molecular choreography that regulates STIM1 expression during pigmentation remain completely unexplored. Therefore, the aim of this study was to delineate molecular events that control STIM1 expression during melanogenesis.

We here report that physiological melanogenic stimuli αMSH regulates STIM1 expression at transcriptional level *via* action of MITF. We demonstrate that αMSH treatment enhances both STIM1 expression and SOCE. Downstream of αMSH signaling, we identified a critical role of MITF transcription factor in regulating STIM1 expression during melanogenesis in B16 cells and primary human melanocytes. Our data reveal that MITF silencing leads to decrease in STIM1 expression as well as SOCE. Similarly, we observe an increase in both STIM1 expression and SOCE upon MITF overexpression. Further, we identified MITF-binding sites on STIM1 promoter and validated MITF binding on STIM1 promoter by performing chromatin immunoprecipitation (ChIP) assay. Moreover, we generated a number of truncated STIM1 promoters and characterized the precise MITF-binding sites on STIM1 promoter. Finally, we evaluated publicly available RNA-seq datasets and observed a positive correlation between STIM1 and MITF expression in sun-exposed tanned human skin. This unbiased big data analysis implies the physiological significance of STIM1 regulation by MITF. Collectively, our study has established MITF as a novel positive transcriptional regulator of STIM1 in melanocytes.

## Results

### STIM1 expression and activity increases with melanogenesis

**Figure 1:**
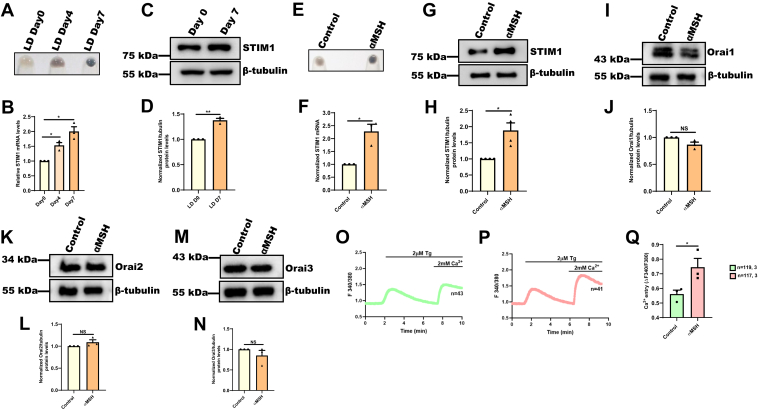
**STIM1 expression and activity increases with melanogenesis.***A*, representative B16 cell pellet pictures of LD day 0, LD day 4, and LD day 7. *B*, qRT–PCR analysis showing increase in STIM1 mRNA expression with pigmentation in B16 LD model (N = 3). *C*, representative Western blot showing an increase in STIM1 protein expression on LD day 7 in comparison with day 0 (N = 3). *D*, densitometric quantitation showing increase in STIM1 protein levels on LD day 7 in comparison with day 0 (N = 3). *E*, representative pellet pictures upon αMSH treatment in B16 cells as compared to control (N = 3). *F*, qRT–PCR analysis showing increase in STIM1 mRNA expression in B16 cells upon αMSH treatment (N = 3). *G*, representative Western blot showing STIM1 protein levels in B16 cells upon 1 μM αMSH treatment for 48 h as compared to vehicle control (N = 4). *H*, densitometric quantitation showing increase in STIM1 protein levels upon αMSH treatment (N = 4). *I*, representative Western blot showing Orai1 protein levels upon αMSH treatment in B16 cells as compared to vehicle control (N = 3). *J*, densitometric quantitation showing Orai1 protein levels upon αMSH treatment (N = 3). *K*, representative Western blot showing Orai2 protein levels upon αMSH treatment in B16 cells as compared to control (N = 3). *L*, densitometric quantitation showing Orai2 protein levels upon αMSH treatment (N = 3). *M*, representative Western blot showing Orai3 protein levels upon αMSH treatment in B16 cells as compared to control (N = 3). *N*, densitometric quantitation showing Orai3 protein levels upon αMSH treatment (N = 3). *O*, representative Ca^2+^ imaging trace of vehicle control where “n = 43” denotes the number of cells in that particular trace. Cells were stimulated with 2 μM thapsigargin (Tg) in Ca^2+^-free buffer followed by restoration of 2 mM extracellular Ca^2+^. *P*, representative Ca^2+^ imaging trace of αMSH treatment where “n = 41” denotes the number of cells in that particular trace. Cells were stimulated with 2 μM thapsigargin (Tg) in Ca^2+^-free buffer followed by restoration of 2 mM extracellular Ca^2+^. *Q*, the extent of SOCE was calculated from 119 Control and 117 αMSH-treated B16 cells, which were imaged from three independent experiments (“n = x, y” where “x” denotes total number of cells imaged and “y” denotes number of traces recorded). Data presented are mean ± S.E.M. For statistical analysis, one sample *t* test was performed for panels *B*, *D*, *F*, *H*, *J*, *L*, and *N*, and unpaired student’s *t* test was performed for panel *Q* using GraphPad Prism software. Here, NS means nonsignificant; ∗*p* <0.05 and ∗∗*p* < 0.01. αMSH, α-Melanocyte Stimulating Hormone; LD, low-density; SOCE, store-operated Ca2+ entry; STIM1, Stromal Interaction Molecule1.

We have established a B16 (mouse melanoma cell line)-based LD culturing–induced pigmentation model (9). This model closely recapitulates the melanogenic pathways and signaling cascades operating in primary human melanocytes (9, 24, 25). The LD-pigmentation model leads to a gradual increase in pigmentation over a 7-day period wherein the day-zero (D0) cells are depigmented; by day four (D4), the pigmentation machinery becomes activated and cells become completely pigmented by day seven (D7) (Fig. 1*A*). To evaluate the association of STIM1 expression with pigmentation levels, we examined STIM1 mRNA and protein levels in the LD pigmentation model. We observed that as B16 cells pigment in the LD model, both mRNA (Fig. 1*B*) and protein expression of STIM1 increases significantly (Fig. 1, *C* and *D*). This suggests that STIM1 expression is positively related to pigmentation levels in B16 cells.

After observing increased expression of STIM1 in B16 LD pigmentation model, we next asked the question whether this increase in STIM1 expression occurs in response to physiological melanogenic stimuli such as αMSH (26). Using αMSH, we induced pigmentation in B16 high density (HD) cells. As presented in Figure 1*E*, 1 μM αMSH treatment for 48 h induced pigmentation in B16 cells. Next, we evaluated mRNA and protein expression of STIM1 upon αMSH treatment in B16 cells. Interestingly, we observed around two fold increase in STIM1 expression both at mRNA (Fig. 1*F*) and protein levels (Fig. 1, *G* and *H*) in response to αMSH-induced pigmentation in B16 cells. This suggests that STIM1 expression is positively associated with LD culturing–induced pigmentation as well as αMSH-mediated physiological pigmentation.

We next examined the levels of Orai channel proteins (Orai1/2/3) upon αMSH treatment as they act as cognate partners of STIM1 for driving SOCE. We observed that αMSH does not enhance the expression of Orai channels (Fig. 1, *I*–*N*). We then asked the question if the increase in STIM1 expression upon αMSH treatment is enough to enhance SOCE. We used standard thapsigargin (Tg)-activated SOCE protocol (27). We gave Tg (2 μM) treatment in the absence of extracellular Ca^2+^, which leads to depletion of ER Ca^2+^ stores by blocking SERCA channels. We induced ER Ca^2+^ release with Tg and waited until the cytosolic Ca^2+^ levels reached back to the baseline. Subsequently, we added Ca^2+^ (2 mM) in the bath solution so that it may be taken up by cells through SOCE. We used ratiometric Fura-2AM dye for measuring changes in the cellular Ca^2+^ levels. We performed these experiments multiple times and analyzed Ca^2+^ imaging data from around 120 cells/condition from three independent experiments. We quantitated SOCE by determining the increase in the ratio of fluorescence emission intensity (acquired with excitation of Fura-2AM at 340 nm and 380 nm) upon addition of Ca^2+^. As presented in Figure 1O-Q, αMSH treatment for 48 h leads to a significant increase in the SOCE, suggesting that αMSH-induced increase in STIM1 expression is enough to enhance SOCE in B16 cells. Interestingly, we had previously observed a similar increase in SOCE during LD pigmentation as well (9). Overall, the data suggests that both STIM1 expression and activity is enhanced upon αMSH treatment.

### MITF positively regulates STIM1 promoter activity in melanocytes

**Figure 2:**
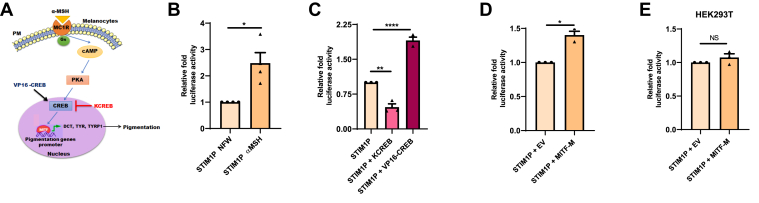
**MITF regulates STIM1 promoter activity in melanocytes.***A*, schematic representation of the αMSH-induced melanogenesis *via* cAMP-PKA-CREB-MITF signaling axis. *B*, normalized luciferase activity of STIM1 promoter in B16 cells upon 1 μM αMSH treatment for 48 h (N = 4). *C*, normalized luciferase activity of STIM1 promoter upon overexpression of either KCREB or VP16-CREB in B16 cells (N = 3). *D*, normalized luciferase activity of STIM1 promoter upon MITF overexpression in B16 cells (N = 3). *E*, normalized luciferase activity of STIM1 promoter upon MITF overexpression in HEK293T cells (N = 3). Data presented are mean ± S.E.M. For statistical analysis, one sample *t* test was performed for panels *B*, *D*, and *E*, while one-way ANOVA followed by Tukey’s post hoc test was performed for panel *C*. F and *p* values for panel *C* are F (2, 6) = 153.9 and *p* < 0.0001, respectively. Here, NS means nonsignificant; ∗*p* <0.05; ∗∗*p* < 0.01, and ∗∗∗∗*p* < 0.0001. CREB, cAMP response element binding protein; cAMP, cyclic AMP; MITF, Microphthalmia-associated transcription factor; STIM1, Stromal Interaction Molecule1; αMSH, α-Melanocyte Stimulating Hormone.

αMSH is a physiological stimulator of melanogenesis (26) and it induces melanogenesis *via* cAMP-PKA-CREB-MITF signaling pathway (19, 20, 21). Please refer to Figure 2*A* for the diagrammatic illustration of signaling module working downstream of αMSH for stimulating melanogenesis. We observed that αMSH treatment leads to augmentation of STIM1 expression (both at mRNA and protein levels) in B16 cells (Fig. 1, *E*–*H*). Increase in STIM1 expression at mRNA levels upon αMSH treatment points towards plausible STIM1 transcriptional upregulation downstream of αMSH. Therefore, we next evaluated STIM1 promoter activity upon αMSH treatment. We generated a human STIM1 promoter clone (624 bp region) spanning −608 bp to +16 bp around STIM1 transcription start site. The promoter was cloned into pGL4.23 luciferase reporter vector after restriction digestion at KpnI/HindIII sites. The promoter cloning was confirmed with double digestions and further validated by sequencing. We examined STIM1 promoter activity upon 1 μM αMSH treatment for 48 h. We found around 2.5-fold increase in STIM1 promoter activity upon αMSH treatment as compared to vehicle (nuclease free water)-treated cells (Fig. 2*B*). This data suggests that αMSH treatment enhances STIM1 promoter activity. αMSH treatment leads to activation of CREB downstream of cAMP-PKA signaling, which is a key transcriptional regulator of MITF. We therefore evaluated the role of CREB in regulating STIM1 promoter activity. We overexpressed dominant negative CREB (KCREB) (28) and constitutively active CREB (VP16-CREB) (29) for understanding the role of CREB in STIM1 promoter activity. We observed that ectopic expression of KCREB leads to decrease in STIM1 promoter activity while that of VP16-CREB results in enhancement of STIM1 promoter activity (Fig. 2*C*). Since in melanocytes, CREB typically mediates its effects *via* MITF, we next investigated STIM1 promoter activity upon MITF-M (melanocyte-specific isoform of MITF) (30) overexpression. Our analysis show an increase in STIM1 promoter activity upon ectopic expression of MITF-M (Fig. 2*D*). Further, in order to understand that whether STIM1 regulation by MITF-M is specific to melanocytes, we overexpressed MITF-M in nonpigmenting HEK293T cells. Interestingly, overexpression of MITF-M in HEK293T cells did not show any change in STIM1 promoter activity (Fig. 2*E*). This suggested that transcriptional regulation of STIM1 by MITF is a cell type–specific phenomenon.

### MITF silencing decreases STIM1 expression and activity

**Figure 3:**
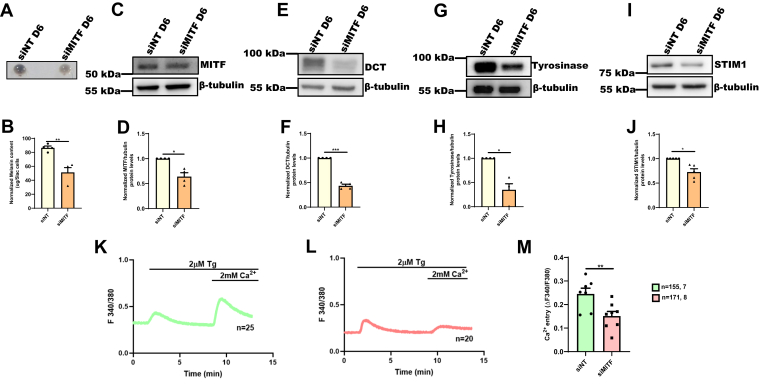
**MITF silencing decreases STIM1 expression and activity.***A*, representative pellet pictures of siNonTargeting (siNT) control and siMITF on LD day 6 (N = 4). *B*, melanin content estimation of siNT and siMITF B16 cells on LD day 6 (N = 4). *C*, representative Western blot confirming siRNA-based silencing of MITF on LD day 6 B16 cells (N = 4). *D*, densitometric quantitation showing MITF levels on LD day 6 in siNT control and siMITF condition (N = 4). *E*, representative Western blot showing expression of DCT on LD day 6 upon MITF silencing as compared to nontargeting control (N = 4). *F*, densitometric quantitation showing DCT levels on LD day 6 in siNT control and siMITF condition (N = 4). *G*, representative Western blot showing expression of Tyrosinase on LD day 6 upon MITF silencing as compared to nontargeting control (N = 4). *H*, densitometric quantitation showing Tyrosinase levels on LD day 6 in siNT control and siMITF condition (N = 4). *I*, Western blot analysis for STIM1 expression on LD day 6 upon MITF silencing as compared to nontargeting control B16 cells (N = 5). *J*, densitometric quantitation showing STIM1 levels on LD day 6 in siNT control and siMITF (N = 5). *K*, representative Ca^2+^ imaging trace of siNT where “n = 25” denotes the number of cells in that particular trace. Cells were stimulated with 2 μM thapsigargin (Tg) in Ca^2+^-free buffer followed by restoration of 2 mM extracellular Ca^2+^. *L*, representative Ca^2+^ imaging trace of siMITF where “n = 20” denotes the number of cells in that particular trace. Cells were stimulated with 2 μM thapsigargin (Tg) in Ca^2+^-free buffer followed by restoration of 2 mM extracellular Ca^2+^. *M*, the extent of SOCE was calculated from 155 siNT and 171 siMITF B16 cells, which were imaged from 7 to 8 independent experiments (“n = x, y” where “x” denotes total number of cells imaged and “y” denotes number of traces recorded). Data presented are mean ± S.E.M. For statistical analysis, one sample *t* test was performed for panels *D*, *F*, *H*, and *J*, and unpaired student’s *t* test was performed for panels *B* and *M*. Here, ∗ *p* < 0.05; ∗∗ *p* < 0.01; ∗∗∗ *p* < 0.001. DCT, Dopachrome Tautomerase; LD, low-density; MITF, Microphthalmia-associated transcription factor; NT, nontargeting; SOCE, store-operated Ca2+ entry; STIM1, Stromal Interaction Molecule1.

Upon observing transcriptional regulation of STIM1 by MITF, we next asked what happens to STIM1 expression and activity upon MITF silencing. For examining the role of MITF in regulating STIM1 expression in melanocytes, we utilized B16 LD pigmentation model. We performed siRNA-mediated silencing of MITF on day 3 of B16 LD pigmentation model and analyzed the pigmentation phenotype on LD day 6 (Fig. 3*A*). As expected, we observed a substantial decrease in pigmentation phenotype upon MITF silencing in comparison to control nontargeting (NT) siRNA condition, which was evident in B16 LD day 6 pellet pictures (Fig. 3*A*). We quantitated the decrease in the melanogenesis upon MITF silencing by performing melanin content assay, which showed around 45% inhibition of melanin synthesis upon MITF silencing (Fig. 3*B*). We next evaluated MITF expression in the siMITF B16 cells and observed around 40% reduction in MITF levels (Fig. 3, *C* and *D*).We then analyzed the expression of melanogenic enzymes known to be transcriptionally regulated by MITF, that is, tyrosinase-related protein 2/Dopachrome Tautomerase (DCT) and tyrosinase in the siNT and siMITF cells. We observed that MITF silencing results in significant reduction in the expression of DCT and tyrosinase (Fig. 3, *E*–*H*). Further, we observed about 35% decrease in STIM1 expression upon MITF silencing in comparison to control cells (Fig. 3, *I* and *J*). These data substantiate the critical role of MITF in regulating STIM1 expression during melanogenesis. To rule out the possibility of off target effects of MITF siRNA, we performed similar experiments with an independent set of siRNAs procured from a different manufacturer. As expected, we obtained similar results with this independent MITF siRNA, that is, MITF knockdown decreases STIM1 expression in B16 cells (Fig. S1, *A*–*J*).

In order to understand whether MITF regulates STIM1 function, that is, SOCE in B16 cells, we performed live cell Ca^2+^ imaging in siNT and siMITF cells. As discussed above, we used standard Tg-activated SOCE protocol (27). We performed live cell experiments multiple times and analyzed Ca^2+^ imaging data from over 150 cells/condition. We quantitated SOCE by determining the increase in the ratio of fluorescence emission intensity (acquired with excitation of Fura-2AM at 340 nm and 380 nm) upon addition of Ca^2+^. As expected, we observed a significant decrease in SOCE in MITF-silenced cells as compared to siNT-transfected control cells (Fig. 3, *K*–*M*). Moreover, we performed similar Ca^2+^ imaging experiments with the independent set of siRNAs and observed comparable results, that is, MITF silencing significantly decreases SOCE in B16 cells (Fig. S1, *K*–*M*). Taken together, our live cell Ca^2+^ imaging experiments clearly demonstrate that MITF regulates STIM1 function in melanocytes.

### MITF overexpression enhances STIM1 expression and activity

**Figure 4:**
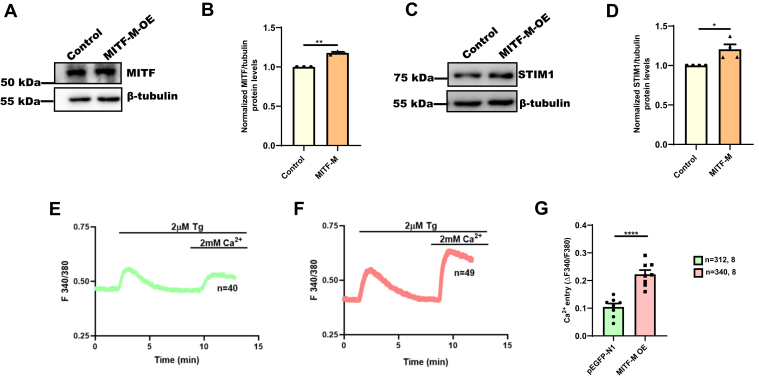
**MITF overexpression enhances STIM1 expression and activity.***A*, Western blot analysis for examining MITF protein levels upon MITF-M overexpression in B16 cells (N = 3). *B*, densitometric quantitation of MITF levels upon MITF-M overexpression as compared to control (N = 3). *C*, Western blot analysis for examining STIM1 protein levels upon MITF-M overexpression in B16 cells (N = 4). *D*, densitometric quantitation of STIM1 levels upon MITF-M overexpression as compared to control (N = 4). *E*, representative Ca^2+^ imaging trace of control plasmid pEGFP-N1, where “n = 40” denotes the number of cells in that particular trace. Cells were stimulated with 2 μM thapsigargin (Tg) in Ca^2+^-free buffer followed by restoration of 2 mM extracellular Ca^2+^. *F*, representative Ca^2+^ imaging trace of MITF-M overexpression, where “n = 49” denotes the number of cells in that particular trace. Cells were stimulated with 2 μM thapsigargin (Tg) in Ca^2+^-free buffer followed by restoration of 2 mM extracellular Ca^2+^. *G*, the extent of SOCE was calculated from 312 control plasmid pEGFP-N1 and 340 MITF-M–overexpressed B16 cells, which were imaged from eight independent experiments (“n = x, y” where “x” denotes total number of cells imaged and “y” denotes number of traces recorded). Data presented are mean ± S.E.M. For statistical analysis, one sample *t* test was performed for panel *B* and *D*, and unpaired student’s *t* test was performed for panel *G*. Here, ∗*p* < 0.05; ∗∗*p* < 0.01; ∗∗∗∗*p* < 0.0001. MITF, Microphthalmia-associated transcription factor; STIM1, Stromal Interaction Molecule1; SOCE, store-operated Ca2+ entry.

Our data demonstrate that MITF silencing leads to decrease in STIM1 expression and activity in melanocytes. To further corroborate the role of MITF in STIM1 regulation, we studied the effect of MITF-M overexpression on STIM1 expression and activity. We exogenously expressed MITF-M in B16 cells (Fig. 4*A*) and then examined the expression of STIM1. We observed that MITF levels were increased to 1.2 fold upon its ectopic expression in B16 cells (Fig. 4*B*). Further, this led to a concomitant increase in STIM1 protein expression (Fig. 4, *C* and *D*). We next investigated STIM1 activity upon MITF-M overexpression using live cell Ca^2+^ imaging experiments. We overexpressed MITF-M in B16 cells and examined SOCE. We performed Ca^2+^ imaging experiments upon MITF-M overexpression in over 300 cells/condition. As presented in Figure 4, *E*–*G*, B16 cells overexpressing MITF-M (Fig. 4*F*) showed higher SOCE in comparisons to control pEGFP-N1 (Fig. 4*E*). Taken together, our data clearly highlights a critical role of MITF in regulating STIM1 expression and function.

### Identification of MITF-binding sites on STIM1 promoter

**Figure 5:**
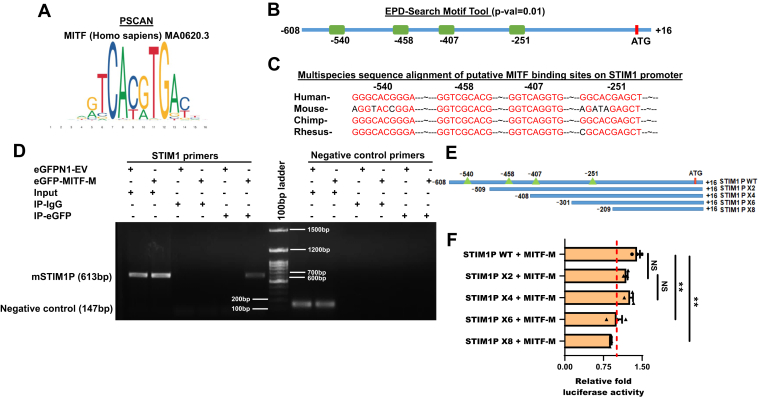
**MITF binds on STIM1 promoter and regulate STIM1 promoter activity.***A*, position weight matrix for human MITF consensus binding sequence. *B*, identification of putative MITF-binding sites on the STIM1 promoter using the EPD-Search Motif Tool at p-val cut-off of 0.01. *C*, bioinformatic characterization of conserved MITF-binding sites on the cross species alignment of the STIM1 promoter using the ContraV3 transcription factor binding analysis tool. *D*, chromatin immunoprecipitation assay in B16 cells with overexpression of eGFP-MITF-M or vector control. Crosslinked and sonicated chromatin from both conditions was immunoprecipitated using specific GFP antibody or control IgG. Immunoprecipitated DNA was amplified using primers specific to either mouse STIM1 promoter aligned to human STIM1 promoter or negative control region. PCR products were visualized on agarose gel (N = 3). *E*, the cartoon representing potential MITF-binding sites in WT hSTIM1 promoter and different truncations in hSTIM1 promoter (*green triangles* represent four putative MITF-binding sites within the 624bp-cloned region of the STIM1 promoter and *red bar* represent ATG start site). *F*, normalized luciferase activity of WT STIM1 promoter and different truncated STIM1 promoters upon overexpression of MITF-M in B16 cells (N = 3). Data presented are mean ± S.E.M. One-way ANOVA followed by Tukey’s post hoc test was performed for panel *F*. *F* and *p* values for panel *F* are *F* = 11.10 and *p* = 0.0011, respectively. Here, NS means nonsignificant and ∗∗*p* < 0.01. EPD, Eukaryotic Proteome Database; MITF, Microphthalmia-associated transcription factor; STIM1, Stromal Interaction Molecule1.

As our data clearly show that MITF regulates STIM1 expression, we now wanted to investigate the molecular details of this regulation. To delineate molecular mechanisms controlling STIM1 expression during melanogenesis, we analyzed STIM1 promoter for potential binding sites for MITF. We performed extensive bioinformatics analysis of the STIM1 promoter using three different analysis tools namely PSCAN (31), Eukaryotic promoter database (EPD-)Search Motif Tool (https://epd.epfl.ch//index.php), and ContraV3 (32). PSCAN was used to identify all putative TF-binding sites present in the STIM1 promoter. This was performed using JASPAR Core 2020 nonredundant transcription factor position weight matrix database (Fig. 5*A*). The STIM1 promoter was also analyzed for presence of MITF-binding sites using the EPD-Search Motif Tool. Human STIM1 promoter sequence was retrieved and analyzed for the presence of probable MITF-binding sites at a *p*-value cut-off of *p* = 0.01. Further, we evaluated human STIM1 promoter sequence for the presence of putative MITF-binding sites by the ContraV3 analysis tool with core value = 0.90 and similarity matrix value = 0.75. Importantly, all three tools showed the presence of four putative MITF-binding sites on the STIM1 promoter through utilization of different algorithms and TF position weight matrix combinations. Based on this extensive analysis, we focused on the four potential MITF-binding sites in the STIM1 promoter located at -540 bp, -458 bp, -407 bp, and -251 bp before transcription start site (Fig. 5*B*). Further, multi-species alignment of the STIM1 promoter demonstrated that these putative sites are largely conserved across multiple mammalian species (Fig. 5*C*). Collectively, our thorough bioinformatics analysis led to identification of multiple prospective MITF-binding sites in the STIM1 promoter.

### MITF binds on STIM1 promoter and regulates STIM1 promoter activity

To validate our *in-silico* findings, we performed ChIP assay. We overexpressed pEGFP-MITF-M or empty vector control (pEGFP-EV) in B16 cells to examine if MITF-M could directly bind to the STIM1 promoter (Fig. 5*D*). We crosslinked and sonicated chromatin from both conditions and immunoprecipitated them with either GFP antibody or IgG control. The immunoprecipitated DNA was amplified using primers specific to either mouse STIM1 promoter or negative control DNA region with no putative MITF-binding site. Essentially, PCR was performed for six experimental conditions as follows: input DNA samples for pEGFP-EV and pEGFP-MITF-M overexpression conditions consisting of crosslinked, sonicated chromatin that was not subjected to IP (Fig. 5*D* lane 1 and 2 of both STIM1 and negative control condition). IgG conditions, consisting of crosslinked, sonicated chromatin subjected to mock IP with isotype control IgG antibody for pEGFP-EV and pEGFP-MITF-M overexpression conditions (Fig. 5*D* lane 3 and 4 of both STIM1 and negative control condition). Finally, IP conditions consisting of crosslinked, sonicated chromatin subjected to IP with specific anti-GFP antibody (Fig. 5*D* lane 5 and 6 of both STIM1 and negative control condition). All six conditions were probed for STIM1 promoter region and negative control region using primers specific to the mouse STIM1 core promoter and negative control DNA region, respectively. Excitingly, in line with our bioinformatics data, we observed binding of MITF-M only on the endogenous STIM1 promoter (lane 6 of STIM1 condition) but not on negative control DNA region, thereby suggesting that MITF could physically associate with the STIM1 core promoter (Fig. 5*D*). Next, we were interested to identify the exact binding site critical for MITF-mediated STIM1 promoter regulation. Our bioinformatics analysis demonstrated presence of four putative MITF-binding sites within the 624bp-cloned region of the STIM1 promoter. To determine the key MITF-binding site/s, we generated truncated STIM1 promoter fragments using WT STIM1 promoter as template. Truncated promoter fragments with successive 100 bp deletions as depicted schematically in Figure 5*E* were then individually cloned into luciferase reporter vector and designated as X2, X4, X6, and X8, respectively. Subsequently, we performed luciferase assay with all truncated promoter fragments with overexpression of MITF-M in B16 cells. We then evaluated normalized (normalized to respective empty vector control) luciferase activity of WT STIM1 promoter and different truncated STIM1 promoters upon ectopic expression of MITF-M.

In these assays, we observed a substantial but statistically nonsignificant reduction in luciferase activity of X2 promoter fragment suggesting that -540 bp site at least partially contributes to the MITF-mediated STIM1 regulation (Fig. 5*F*). Next, luciferase assays with X4 promoter fragment did not further decrease the STIM1 promoter activity thereby implicating that -458 bp site does not contribute to MITF-driven STIM1 promoter activity. Interestingly, luciferase assays with X6 promoter fragment completely abolished MITF-driven STIM1 promoter activity (Fig. 5*F*). This suggests that -407 bp site significantly contributes to the MITF-mediated STIM1 regulation. Intriguingly, luciferase assays with X8 promoter fragment led to a marginal but statistically significant decrease in the relative luciferase activity (Fig. 5*F*). A possible reason for this observation could be that along with predicted MITF-binding site (-251 bp), the -301 bp to -209 bp region might contain some other regulatory elements binding sites. Therefore, their deletion results in decrease in luciferase activity of this truncated STIM1 promoter. In any case, our data suggests that -540 bp and -407 bp MITF-binding sites contribute to regulation of STIM1 promoter activity. Further, our data implicates that -407 bp MITF-binding site is the most critical regulator of MITF-driven STIM1 expression in melanocytes. These observations further demonstrate that STIM1 is in fact a MITF target gene and that MITF transcriptionally regulates STIM1 expression during the process of pigmentation.

### MITF regulates STIM1 expression downstream of αMSH

**Figure 6:**
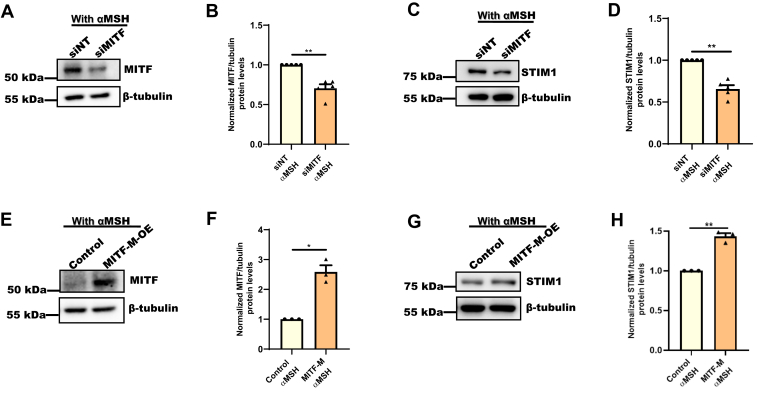
**MITF regulates STIM1 expression upon physiological stimuli.***A*, Western blot analysis for MITF expression upon MITF silencing in presence of αMSH treatment (1 μM for 48 h) in B16 cells (N = 5). *B*, densitometric quantitation of MITF levels in siNT control and siMITF B16 cells in presence of αMSH treatment (N = 5). *C*, Western blot analysis for STIM1 expression upon MITF silencing in presence of αMSH treatment in B16 cells (N = 5). *D*, densitometric quantitation of STIM1 levels in siNT control and siMITF B16 cells in presence of αMSH treatment (N = 5). *E*, Western blot analysis for MITF expression upon MITF overexpression in presence of αMSH treatment in B16 cells (N = 3). *F*, densitometric quantitation of MITF levels in control and MITF-overexpressing B16 cells in presence of αMSH treatment (N = 3). *G*, Western blot analysis for STIM1 expression upon MITF overexpression in presence of αMSH treatment in B16 cells (N = 3). *H*, densitometric quantitation of STIM1 levels in control and MITF-overexpressing B16 cells in presence of αMSH treatment (N = 3). Data presented are mean ± S.E.M. For statistical analysis, one sample *t* test was performed for panel *B*, *D*, *F*, and *H* using GraphPad Prism software. Here, ∗*p* < 0.05; ∗∗*p* < 0.01. αMSH, α-Melanocyte Stimulating Hormone; MITF, Microphthalmia-associated transcription factor; NT, nontargeting; STIM1, Stromal Interaction Molecule1.

We observed that αMSH-induced pigmentation is associated with increase in STIM1 levels (Fig. 1, *E*–*H*) and MITF regulates STIM1 expression in LD pigmentation (Figs. 3 and S1). Next, we were interested in investigating the role of MITF in αMSH-mediated rise in STIM1 expression. To examine this, we silenced MITF in nonpigmenting HD B16 cells and then evaluated STIM1 expression in response to αMSH stimulation (Fig. 6). We first confirmed siRNA-mediated MITF knockdown in HD B16 cells treated with 1 μM αMSH for 48 h (Fig. 6*A*). We observed close to 40% decrease in MITF levels in siMITF condition in comparison to siNT control condition (Fig. 6*B*). Further, we found that in presence of αMSH, STIM1 expression was significantly reduced in siMITF cells as compared to siNT cells (Fig. 6, *C* and *D*). To further corroborate the role of MITF in STIM1 regulation downstream of αMSH, we studied the effect of MITF-M overexpression on STIM1 expression in response to αMSH stimulation. We overexpressed MITF-M in B16 cells, then treated these cells with 1 μM αMSH for 48 h. We observed a significant increase in MITF levels upon MITF-M overexpression in the presence of αMSH treatment (Fig. 6, *E* and *F*). Next, we examined the expression of STIM1 in response to MITF-M overexpression along with αMSH stimulation (Fig. 6*G*). We observed a 1.5-fold increase in STIM1 protein levels in MITF-M overexpressing cells as compared to control cells (Fig. 6, *G* and *H*). Taken together, this data clearly demonstrates that MITF-M regulates STIM1 expression downstream of αMSH stimulation.

### MITF regulates STIM1 expression in primary human melanocytes

**Figure 7:**
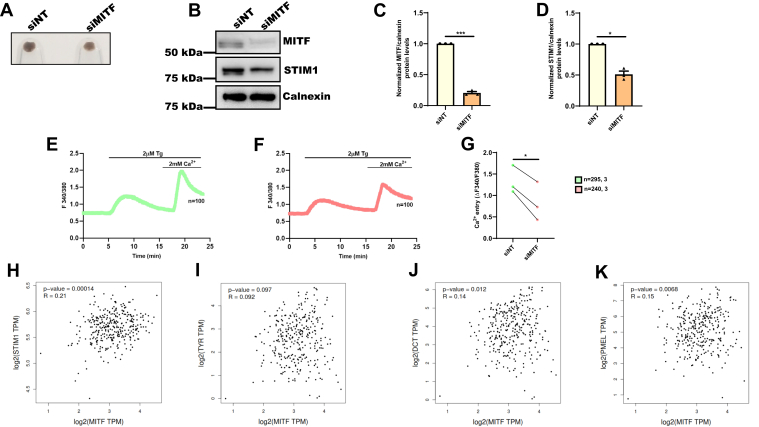
**MITF regulates STIM1 expression in primary human melanocytes.***A*, pellet pictures of primary human melanocytes transfected with either siNT or siMITF (N = 3). *B*, Western blot analysis for MITF and STIM1 expression upon MITF silencing as compared to nontargeting control siRNA in primary human melanocytes (N = 3). *C*, densitometric quantitation of MITF levels in siNT and siMITF condition in primary human melanocytes (N = 3) *D*, densitometric quantitation of STIM1 levels in siNT and siMITF condition in primary human melanocytes (N = 3). *E*, representative Ca^2+^ imaging trace of siNT where “n = 100” denotes the number of cells in that particular trace stimulated with 2 μM thapsigargin (Tg) in Ca^2+^-free buffer followed by restoration of 2 mM extracellular Ca^2+^. *F*, representative Ca^2+^ imaging trace of siMITF where “n = 100” denotes the number of cells in that particular trace stimulated with 2 μM thapsigargin (Tg) in Ca^2+^-free buffer followed by restoration of 2 mM extracellular Ca^2+^. *G*, the extent of SOCE was calculated from 295 siNT and 240 siMITF primary human melanocytes, which were imaged from three independent experiments (“n = x, y” where “x” denotes total number of cells imaged and “y” denotes number of traces recorded). *H*–*K*, dot plots showing mRNA expression correlation analysis between MITF and STIM1/TYR/DCT or Gp100 in human sun-exposed skin tissue samples. “R” signifies value of Pearson’s correlation coefficient for each correlation analysis. Data presented are mean ± S.E.M. For statistical analysis, one sample *t* test was performed for panel *C* and *D*, and paired student’s *t* test was performed for panel *G* using GraphPad Prism software. Here, ∗*p* < 0.05; ∗∗∗*p* < 0.001. DCT, Dopachrome Tautomerase; MITF, Microphthalmia-associated transcription factor; STIM1, Stromal Interaction Molecule1; SOCE, store-operated Ca2+ entry.

To further strengthen our data, we examined the role of MITF in regulating STIM1 expression in primary human melanocytes. By utilizing siRNAs targeting human MITF, we silenced it in primary human melanocytes. We observed that MITF silencing in primary human melanocytes led to a significant decrease in melanogenesis as evident in pellet pictures (Fig. 7*A*). We observed around 80% decrease in the MITF expression in siMITF in comparison to siNT-transfected primary human melanocytes cells (Fig. 7, *B* and *C*). Further, our data shows about 50% decrease in STIM1 expression upon MITF silencing in primary human melanocytes (Fig. 7, *B* and *D*). Next, we investigated the effect of MITF knockdown on STIM1 function in primary human melanocytes. We analyzed SOCE in primary human melanocytes transfected with either NT control siRNA (siNT) or siRNA targeting human MITF by performing live cell Ca^2+^ imaging as discussed in earlier sections. As observed in B16 cells, MITF knockdown in primary human melanocytes resulted in a significant reduction of SOCE in these cells as well (Fig. 7, *E*–*G*). Taken together, our data demonstrates that MITF is a key regulator of STIM1 expression and activity in primary human melanocytes.

Finally, we analyzed publicly available RNA-seq datasets from sun-exposed human skin tissue samples using the GEPIA2 analysis tool (http://gepia2.cancer-pku.cn/#index) (33) to examine the correlation between STIM1 and MITF mRNA expression during melanogenesis. Excitingly, STIM1 and MITF mRNA expression profile showed statistically significant positive correlation in sun-exposed human skin tissue samples (Fig. 7*H*). To gain confidence in this finding, we next analyzed the expression correlation between bona-fide MITF transcriptional targets in melanocytes such as Tyrosinase, DCT, and Gp100 (Fig. 7, *I*–*K*). Surprisingly, we found that expression correlation between MITF and STIM1 was greater (R = 0.21) (Fig. 7*H*) than the expression correlation between MITF and Tyrosinase (R= 0.092) (Fig. 7*I*), DCT (R= 0.14) (Fig. 7*J*), or Gp100 (R = 0.15) (Fig. 7*K*). This data further implies that MITF-mediated STIM1 regulation is physiologically relevant and plays a crucial role in the process of pigmentation.

In summary, our experimental data from three independent pigmentation models, that is, B16 LD pigmentation model (with two independent sets of siRNAs), αMSH stimulated pigmentation in B16 HD cells, and primary human melanocytes clearly establish that MITF is a critical determinant of STIM1 expression during pigmentation. Further, unbiased big data analysis of tanned human skin underscores the physiological significance of this regulation.

## Discussion

Physiological pigmentation process plays a vital role in protection against harmful UV rays. We recently identified a crucial role of ER calcium sensor, STIM1 in melanogenesis through its interaction with plasma membrane localized adenylyl cyclase 6 (ADCY6) (9). We observed augmented expression of STIM1 (both at mRNA and protein levels) with increase in pigmentation in LD culturing–induced B16 pigmentation model (Fig. 1, *B*–*D*). In this study, we elucidated the molecular mechanism regulating STIM1 expression in melanocytes. We utilized our well-established B16 LD pigmentation model, αMSH-induced pigmentation in B16 HD cells, and primary human melanocytes to delineate the molecular events controlling STIM1 expression in melanocytes. Our data reveal that STIM1 expression is increased in response to key physiological melanogenic stimuli αMSH (Fig. 1, *E*–*H*). Further, αMSH treatment led to a significant increase in SOCE (Fig. 1, *O*–*Q*). Since SOCE is dependent on STIM1-mediated gating and activation of Orai channels (Orai1/2/3), we examined the levels of Orai1, Orai2, and Orai3 upon αMSH treatment. We observed that the expression of Orai channels is not enhanced by αMSH (Fig. 1, *I*–*N*). This suggests that the levels of Orai channels are not limiting in B16 cells and therefore, just the increase in STIM1 expression is enough to augment SOCE in response to αMSH stimulation. Interestingly, in our earlier study, we observed similar results in context of upregulation of SOCE during B16 LD pigmentation (9). We identified that STIM1 and Orai1 are cognate partners that drive SOCE in B16 cells. Further, we found that only STIM1 expression increases, while B16 cells pigment in LD model, the levels of Orai1 remain largely unchanged (9). Collectively, data from two independent pigmentation models imply that melanogenesis is associated with an increase in STIM1 expression.

αMSH regulates melanogenesis by activating cAMP-PKA-CREB-MITF signaling module. The increase in STIM1 expression upon αMSH treatment was associated with 2.5-fold augmentation in STIM1 promoter activity (Fig. 2*B*). This indicates that STIM1 promoter activity is increased during αMSH-induced melanogenesis. Therefore, our work shows that STIM1 expression is enhanced in response to physiological melanogenic stimuli αMSH. Importantly, we earlier showed that STIM1 enhances αMSH-induced melanogenesis independent of Orai channels (9). This suggests that STIM1 levels could be limiting for driving αMSH-mediated melanogenesis. Hence, melanocytes have adopted a signaling cascade, involving MITF transcription factor, to augment STIM1 expression during αMSH-stimulated melanogenesis.

Further, our data demonstrates that CREB, which is a key transcriptional regulator of MITF, increases STIM1 promoter activity. Overexpression of constitutively active CREB (VP16-CREB) significantly enhanced the luciferase activity, whereas dominant negative CREB (KCREB) decreased the luciferase activity of the STIM1 promoter (Fig. 2*C*). Furthermore, overexpression of melanocyte-specific MITF isoform (MITF-M) significantly elevated reporter activity of STIM1 promoter (Fig. 2*D*). Interestingly, overexpression of MITF-M in nonpigmenting HEK293T cells did not show any change in the reporter activity (Fig. 2*E*). This suggests that transcriptional regulation of STIM1 by MITF might be a highly cell type–specific phenomenon, which could be dependent upon the expression of additional transcriptional coactivators/regulators.

In order to understand the molecular choreography driving increase in STIM1 expression, we analyzed STIM1 promoter for potential MITF-binding sites. Our bioinformatics analysis demonstrated presence of four putative MITF-binding sites within the 624 bp cloned STIM1 promoter region (Fig. 5*B*), indicating that STIM1 could be a transcriptional target of MITF. Further detailed *in vitro* luciferase assays with truncated STIM1 promoter fragments revealed that two MITF-binding sites (-540 bp and -407 bp) contribute to STIM1 promoter activity (Fig. 5*F*). Although both these sites substantially modulate STIM1 promoter activity, only -407 bp is statistically significant suggesting that -407 bp MITF-binding site plays most critical role in regulating STIM1 transcription. Transcription factors like MITF that contain a basic helix-loop-helix and helix–loop–helix leucine zipper motifs interact with their target genes *via* binding to a consensus DNA sequence constituting a 6 bp CANNTG motif, that is, “E-box” (34). The protein-DNA–binding specificity is provided by the basic region in protein and the key DNA interacting bases along with the flanking region. Most of the MITF-bound sites are CAGGTG “E-box” motifs flanked by A and/or T. This consensus “E-box” sequence is located in the promoter region of the MITF target genes like BCL2, tyrosinase, and cathepsin K (CTSK) (35, 36). Interestingly, the genes involved in pigmentation have a specific “E-box” variant with a flanking T at the 5′ end of the core “E-box” motif. This variant is known as “M-box” (37, 38, 39). An intriguing study revealed that the presence of a T residue flanking an “E-box” motif is a crucial determinant of MITF’s ability to bind DNA. It was shown that MITF could not efficiently bind to genes lacking the flanking T nucleotide (40). Interestingly, in depth analysis of -407 bp MITF-binding site (TCAGGTG) on STIM1 promoter suggests that it is a typical example of “M-box”. Therefore, it is not surprising that -407 bp MITF-binding site plays the most critical role in transcriptional regulation of STIM1.

**Figure 8:**
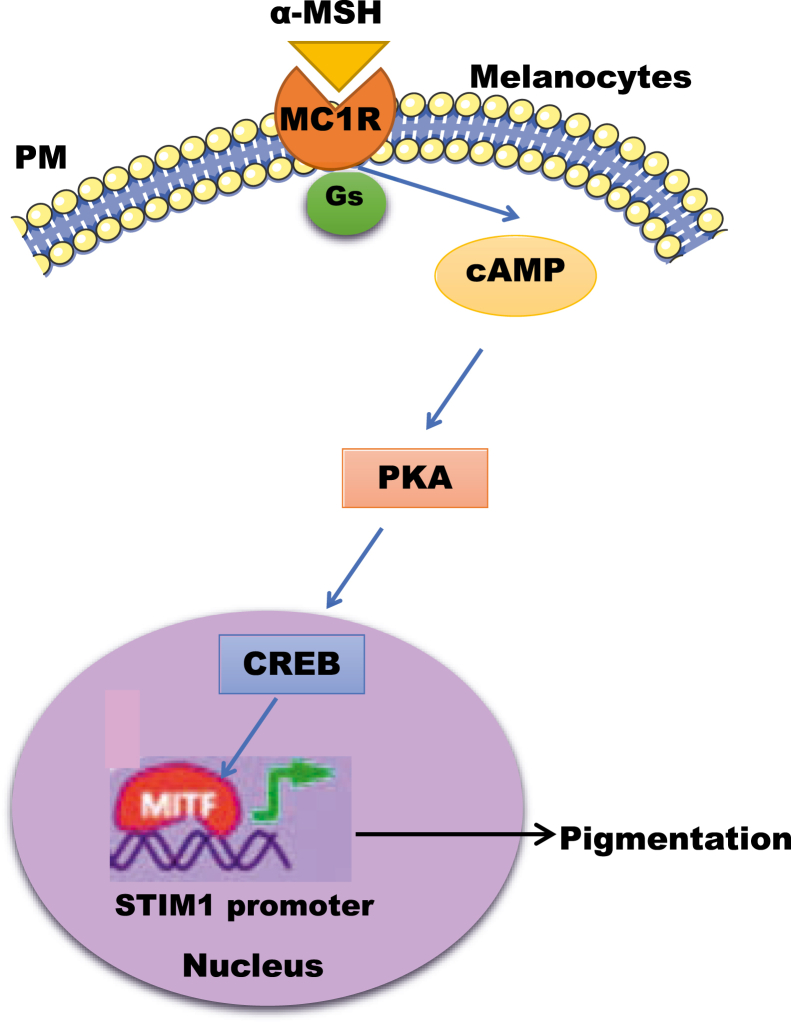
**MITF transcriptionally regulates STIM1 expression during melanogenesis.** Our data demonstrates that downstream of αMSH stimuli, MITF-M regulates STIM1 expression by enhancing transcription of STIM1. We show that MITF-M binds on the STIM1 promoter and thereby drives STIM1 promoter activity and its expression. αMSH, α-Melanocyte Stimulating Hormone; MITF, Microphthalmia-associated transcription factor; STIM1, Stromal Interaction Molecule1.

Our data demonstrated a critical role for MITF in regulating STIM1 expression in both B16 cells and in primary human melanocytes. Moreover, MITF silencing led to decrease in STIM1 activity, that is, SOCE (Figs. 3 and S1), while MITF-M overexpression showed higher SOCE (Fig. 4). Therefore, we have identified MITF as a critical regulator of both STIM1 expression and activity in melanocytes. Notably, analysis of publicly available RNAseq datasets from sun-exposed tanned human skin samples demonstrate that MITF and STIM1 expression is positively correlated in these samples (Fig. 7*H*). Interestingly, the extent of correlation between MITF and STIM1 was more significant than the already established transcriptional targets of MITF, that is, Tyrosinase, DCT, and Gp100 (Fig. 7, *I*–*K*). This unbiased big data analysis further corroborate a positive association between MITF and STIM1 during pigmentation. Taken together, our study has characterized MITF as a novel positive regulator of STIM1 during melanogenesis (Fig. 8).

In our earlier study, we had identified that αMSH activates STIM1, thereby enhancing melanogenesis by generating a positive feedback loop (9). We reported that STIM1 *via* this loop connects depletion of ER Ca^2+^ stores to enhancement of cAMP generation. The increase in cAMP activates MITF-mediated transcription of melanogenic genes. Here, we show that along with melanogenic genes, MITF can also regulate STIM1 transcription. This suggests that during αMSH-induced melanogenesis, STIM1 activation can further enhance its own transcription. Interestingly, it was recently reported that STIM1 can regulate MITF nuclear translocation in RBL cells (41). In this study, the authors overexpressed constitutively active disease-associated STIM1 mutants and observed that it leads to enhanced MITF nuclear translocation in comparison to WT STIM1 overexpression (41). Thereby, highlighting that STIM1 activation can induce nuclear translocation of MITF and that in turn may drive MITF-induced signaling cascade in RBL cells. However, in melanocytes, the major isoform of MITF is MITF-M that typically resides within the nucleus only (30, 42). In future, it would be interesting to understand if STIM1 modulates nuclear translocation of MITF in melanocytes and the functional relevance, if any, of such signaling module.

Collectively, our data suggests that during αMSH-mediated melanogenesis, both STIM1 activation as well as its transcriptional upregulation occurs simultaneously. This might have a physiological significance associated with immediate and delayed tanning. αMSH is a key regulator of tanning response, which typically has two phases, immediate and delayed tanning (43). Since αMSH treatment induces STIM1 activation within minutes (9) and enhances STIM1 expression after several hours, it could be possible that STIM1 contributes to both immediate and delayed tanning response mediated by αMSH. Taken together, our work establishes MITF-STIM1 crosstalk as a critical regulator of physiological pigmentation. Future studies aimed at understanding the role of this signaling module in aging-associated hyperpigmentation and other pigmentary disorders would shed light on pathobiological role of MITF-STIM1 signaling axis.

## Experimental procedures

### Cell culture

B16-F10 cells were obtained from ATCC and cultured in Dulbecco’s modified Eagle’s medium (DMEM, Sigma). Trypsin, Dulbecco’s phosphate buffer saline, Versene, fetal bovine serum (FBS), and additional cell culture grade reagents were obtained from Invitrogen. Cells were cultured in DMEM medium supplemented with 10% FBS (heat inactivated) at 60 to 80% confluence and at 5% CO_2_ levels. Neonatal primary human melanocytes were procured from Invitrogen. Cells were grown in Medium 254 supplemented with human melanocyte growth supplement-2 and maintained at 37 °C in a humidified incubator with 5% CO_2_ atmosphere. Cells between passages 3 to 6 were used for experimentation.

### B16 LD pigmentation model

To set up the LD pigmentation model with B16, cells were seeded at 100 cells/cm^2^ in DMEM supplemented with 10% FBS as described earlier (9, 44). Cells were allowed to gradually pigment and the assays were terminated on LD Day6/7.

### αMSH-induced pigmentation assay

In B16 cells, seeded at HD, pigmentation was induced by adding 1 μM αMSH (Sigma-Aldrich, M4135) for 24 (for mRNA work) to 48 h (for protein work).

### qRT-PCR analysis

For mRNA extraction, cells were processed with Qiagen RNeasy kit (Catalog #74106). mRNA was then converted to cDNA using high-capacity cDNA reverse transcription kit from Thermo Fisher Scientific (Catalog #4368814). Real-time PCR reactions were performed using SYBR green in Quant Studio 6 Flex from Applied Biosystems. The data were analyzed with Quant Studio real-time PCR software version 1.3. The expression of STIM1 was normalized to that of the housekeeping gene GAPDH. Primers were designed using Primer3 and checked by the NCBI Primer blast tool. Gene-specific primers were synthesized by Eurofins Genomics India Pvt. Ltd. The details of primers used in this study are provided in Table 1.

**Table 1:** List of qRT-PCR primers used in the study

Identifier	Gene name	Sequence	Species
LCSP 45a	STIM1	TTCCCTCAGTTCCCACTCCA	Mouse
LCSP 45b	STIM1	CCCCACAACTGCTAGGATCG	Mouse
LCSP 67a	GAPDH	AACTGCTTAGCACCCCTGGC	Mouse
LCSP 67b	GAPDH	ATGACCTTGCCCACAGCCTT	Mouse

### Western blotting

Cells were lysed using NP40 lysis buffer supplemented with protease inhibitors. Typically, 50 to 100 μg proteins were subjected to SDS-PAGE (7.5–10%). Proteins from gels were then electrotransferred onto PVDF membranes. After blocking with 5% non-fat dry milk (NFDM) dissolved in Tris-buffered saline containing 0.1% Tween 20 (TTBS), blots were probed overnight at 4 °C, with specific primary antibodies in TTBS containing 2% NFDM. The primary antibodies used were typically procured from Abcam and were used at 1:500 to 1:2000 dilutions. The following day, membranes were incubated for 2 h at room temperature with a horseradish peroxidase–conjugated anti-mouse or anti-rabbit IgG antibody in TTBS containing 2% NFDM. Detection was performed using the enhanced chemiluminescence reagent (ECL Western blotting detection reagents; Amersham Biosciences). Quantification of bands was performed by densitometry using the ImageJ software. The catalog number and company name for the antibodies are provided in Table 2.

**Table 2:** Details of the antibodies used in the study

Antibody	Company	Catalog number
DCT	Abcam	ab74073
Gp100	Abcam	ab137078
β-Tubulin	Abcam	ab21058
STIM1	Abcam	ab108994
MITF	Abcam	ab12039
Calnexin	Abcam	ab22595
Orai1	Abcam	ab86748
Orai2	Abcam	ab180146
Orai3	Abcam	ab254260

### siRNA-based transient transfections

siRNA transfections were performed in T75 flasks on day 3 of the LD pigmentation model as reported earlier (24). Hundred nanomolar of siRNA (smartpool siRNAs from Dharmacon) was added per flask with a 1: 3 V: V ratio of Dharmafect transfection reagent. siRNA and transfection reagent were mixed and incubated over the cells in OptiMEM (Gibco, Waltham) media for 4 to 6 h for achieving optimal transfection efficiency. siRNA transfections in B16 HD cells were performed using smartpool siRNAs from Dharmacon. Further, an independent set of siRNAs was procured from Sigma. siRNAs from both the manufacturers were transfected with same protocol using Dharmafect transfection reagent in B16 cells. Briefly, siRNA and transfection reagent were mixed and incubated over the cells in OptiMEM (Gibco media for 4 to 6 h for achieving optimal transfection efficiency. siRNA transfection in primary melanocytes was done using Nucleofection Kit (Lonza, VPD-1003, U-024 program). Hundred nanomolar of siRNA and 0.5 μg Pmax-GFP plasmid DNA was added per condition (7–10 lac cells). Media was changed after 24 h of transfection. Cells were harvested post 72 h of transfection to capture phenotype and protein expression changes. The siRNAs (smartpool of four individual siRNAs targeting gene of interest) were procured from Dharmacon. The catalog number and target sequence of siRNAs used in the study are included in Table 3.

**Table 3:** Details of the siRNAs used in the study

siRNA (manufacturer)	Catalog number	Target sequence
siNT (Dharmacon)	D-001810-10-20	UGGUUUACAUGUCGACUAA, UGGUUUACAUGUUGUGUGA, UGGUUUACAUGUUUUCUGA, UGGUUUACAUGUUUUCCUA
siMITF (Mouse) (Dharmacon)	L-047441-00-0010	CGAAGAAGAAGAUUUAACA, GGAGCUAGGUACUCUGAUC, GAAGAAAUUUUGGGCUUGA, AGGCAGACCUGACAUGUAC
siMITF (Human) (Dharmacon)	L-008674-00-0010	UGGCUAUGCUUACGCUUAA, AGAACUAGGUACUUUGAUU, AGACGGAGCACACUUGUUA, GAACACACAUUCACGAGCG
MISSION siRNA Universal Negative Control #1 (Sigma) used in Fig. S1.	SIC001-10	MISSION® Universal Negative Control esiRNA are heterologous mixture of siRNA that do not target any gene.
MISSION siMITF (Mouse) (Sigma) used in Fig. S1.	EMNC002561-20	MISSION esiRNA are heterologous mixture of siRNA that target mRNA sequence of the same gene.

### Cloning of STIM1 core promoter and truncated promoter fragments

Human STIM1 promoter sequence was obtained from EPD, which was followed by NCBI-BLAST analysis of the sequence to identify mRNA start site and first codon. Primers were designed to amplify a 624 bp region (−608 to + 16, with respect to start codon) of the STIM1 core promoter. STIM1 core promoter −608 to +16 (STIM1P WT) was amplified from human genomic DNA, isolated using DNeasy Blood and Tissue Kit (69504, Qiagen) as per manufacturer’s protocol. This was followed by PCR amplification of the target region using Phusion High Fidelity Polymerase (F503, Thermo Fisher Scientific), which was further cloned into pGL4.23 luciferase reporter vector (Promega) at the KpnI/HindIII sites. Further truncated promoter fragments X2, X4, X6, and X8 were PCR amplified using STIM1P WT as template and cloned in pGL3basic luciferase and renilla-polyA (Addgene#129046) vector at KpnI/HindIII sites. STIM1P WT was also simultaneously subcloned into pGL3basic luciferase and renilla-polyA vector at KpnI/HindIII sites. All positive clones were verified by restriction digestion and sequencing to confirm their identity. Primers utilized for cloning of all the fragments are listed in Table 4.

**Table 4:** List of primers used for cloning of STIM1 promoters

Promoter	Primers
STIM1P WT	FP-TAAGGTACCGAAGCCGCTGTCCTGG RP-GGCGAAGCTTGGACGCATACATCCATGACTC
X2	FP-TTAGGTACCCTAGGAGGCCCAGGATCC RP- GGCGAAGCTTGGACGCATACATCCATGACTC
X4	FP- AATTGGTACCGTCAGGTGCCCCCTTCTCG RP- GGCGAAGCTTGGACGCATACATCCATGACTC
X6	FP- AAAGGTACCAATCTGCGGAGCTGACAGCA RP- GGCGAAGCTTGGACGCATACATCCATGACTC
X8	FP- AAAGGTACCACCTGAGGAGCCAGCCC RP- GGCGAAGCTTGGACGCATACATCCATGACTC

### *In vitro* luciferase assay

B16 or HEK293T cells were seeded 24 h before transfection at a density of 0.5 × 10^5^ cells/well in 24-well plates. Cells were transfected with STIM1P WT and truncated fragments (X2-X8) along with pEGFP-MITF-M, VP16-CREB, or KCREB as indicated, using Turbofect transfection reagent (R0532, Thermo Fisher Scientific) as per manufacturer’s protocol. Cells were treated with αMSH or NFW for 48 h where indicated. Renilla luciferase control plasmid was utilized for transfection normalization in all experiments. Forty eight hours post transfection, cells were assayed for luciferase activity using the dual luciferase assay kit (E1910, Promega) as per manufacturer’s protocol. Data is representative of three biological replicates with three technical replicates each. VP16-CREB and KCREB plasmids were generously gifted by David Yule, University of Rochester. Human pEGFP-N1-MITF-M plasmid was a gift from Shawn Ferguson (Addgene plasmid # 38131).

### Calcium imaging

Calcium imaging was performed as reported earlier (27, 45). Briefly, cells were cultured on confocal dishes for performing Ca^2+^ imaging. Cells were incubated at 37 °C for 30 min in a culture medium containing 4 μM fura-2AM. After incubation, cells were washed 3 times and bathed in Hepes-buffered saline solution (140 mM NaCl, 1.13 mM MgCl2, 4.7 mM KCl, 2 mM CaCl2, 10 mM D-glucose, and 10 mM Hepes; pH 7.4) for 5 min before Ca^2+^ measurements were made. A digital fluorescence imaging system (Nikon Eclipse Ti2 microscope coupled with CoolLED pE-340 Fura light source and a high speed PCO camera) was used, and fluorescence images of several cells were recorded and analyzed. Fura-2AM was excited alternately at 340 and 380 nm, and the emission signal was captured at 510 nm. Figures showing Ca^2+^ traces are an average from several cells (the number of cells is denoted as “n” on each trace) attached on a single imaging dish. Each experiment was performed at least 3 times and the final data are plotted in the form of bar graphs.

### MITF overexpression experiments

B16 cells were seeded 24 h before transfection at a density of 1.0 × 10^6^ cells/well in 6-well plates. Human pEGFP-N1-MITF-M plasmid (1.5 μg) was overexpressed in B16 cells plated at 60% confluency using Lipofectamine 2000 (Invitrogen, 11668-019). The effect of MITF overexpression was analyzed post 48 to 72 h by performing Western blotting.

### Melanin content assay

Melanin-content assay was performed as described earlier (9, 24). The cells were lysed in 1N NaOH by heating at 80 °C for 2 h and then absorbance was measured at 405 nm. Melanin content was estimated by interpolating the sample readings on the standard melanin curve (μg/ml) obtained with synthetic melanin.

### ChIP assay

B16 cells transfected with pEGFP-MITF-M overexpression plasmid or pEGFPN1-empty vector were harvested by trypsinization. Cells were counted and resuspended in 10 ml 1× PBS; 25 million cells were with fixed with 1% formaldehyde for 7 min at room temperature then quenched with 4M Tris. Cells were then washed with cold 1× PBS and pellet was obtained by centrifugation at 1500 rpm for 5 min at 4 °C. To perform fragmentation of chromatin, cell pellets were thawed on ice and resuspended in 10 ml of Rinse Buffer 1 (50 mM Hepes pH 8, 140 mM NaCl, 1 mM EDTA, 10% glycerol, 0.5% NP-40, 0.25% Triton X-100) and incubated for 10 min on ice. Cells were further resuspended in Rinse Buffer 2 (10 mM Tris pH 8, 1 mM EDTA, 0.5 mM EGTA, 200 mM NaCl) and incubated on ice for 10 min. Finally, cells were resuspended in Shearing buffer (0.1% SDS, 1 mM EDTA, 10 mM Tris pH 8). To perform sonication, five million cells were resuspended in 300 μl of shearing buffer and sonicated using Bioruptor Pico with 30s ON and 30s OFF for 20 cycles at 4 °C. Sonicated chromatin was centrifuged at 16000 rpm for 10 min to remove debris. DNA concentration of the sonicated chromatin was determined and 100 μg of sonicated chromatin was utilized for immunoprecipitation using 2.5 μg GFP antibody or IgG control. Protein A agarose beads were equilibrated with glycerol IP buffer and blocked with 75 ng/μl Herring sperm DNA and 0.1 μg/μl bovine serum albumin. Post IP beads were washed once each with low salt buffer (0.1% SDS, 1% Triton X100, 2 mM EDTA, 20 mM Tris–Cl pH 8, 150 mM NaCl), high salt buffer (0.1% SDS, 1% Triton X100, 2 mM EDTA, 20 mM Tris–Cl pH 8, 500 mM NaCl), LiCl buffer (0.25 M LiCl, 1% NP-40, 1% sodium deoxycholate, 1 mM EDTA, 10 mM Tris–Cl pH 8) and finally with TE buffer. Decrosslinking was performed at 65 °C overnight and DNA was eluted from the beads using Elution Buffer (1% SDS, 100 mM NaHCO3) for 30 min at 30 °C. Isolated DNA was purified using phenol-chloroform extraction and used for PCR using primers specific to either mouse STIM1 core promoter or negative control DNA region with no putative MITF-binding site. Negative control primers were designed to amplify a 147 bp fragment of a gene desert region on mouse chromosome 5 flanked by semaphorin-3d precursor on the 5′ side at 134355 bp and semaphorin-3a precursor on the 3′ at 680156 bp. Gene desert regions are frequently utilized as negative controls in ChIP experiments as they lack coding genes and are not actively transcribed (46). The primers used for ChIP analysis are as follows:

mSTIM1P F- AAACTCGAGAGCCGCTGTCCCGG

mSTIM1P R- GGGAAGCTTCGCACACATCCATGACGG.

Negative Control F-GGCCCTGTAATTGGAATGAGTC.

Negative Control R- CCAAGGTCCAACTACGAGCTT.

### Statistical analysis

All statistical analysis was performed using GraphPad Prism 8 software. All experiments were performed at least 3 times. Data are presented as mean ± SEM, and the student’s *t* test was performed for determining statistical significance between two experimental samples whereas one-way ANOVA was performed for the comparison of three or more samples. Further, Tukey’s post hoc test was used to compare different groups analyzed with one-way ANOVA. A *p*-value < 0.05 was considered as significant and is presented as “∗”; *p*-value < 0.01 is presented as “∗∗”; *p*-value < 0.001 is presented as “∗∗∗”; and *p*-value < 0.0001 is presented as “∗∗∗∗”.

## Data availability

All data is contained within the article and/or supplementary information.

## Conflict of interest

The authors declare that they do not have any conflict of interest with the content of this article.

## References

[1] Smyth J.T., Hwang S.Y., Tomita T., DeHaven W.I., Mercer J.C., Putney J.W. (2010). Activation and regulation of store-operated calcium entry. J. Cell Mol. Med..

[2] Hogan P.G., Rao A. (2015). Store-operated calcium entry: mechanisms and modulation. Biochem. Biophys. Res. Commun..

[3] Berna-Erro A., Woodard G.E., Rosado J.A. (2012). Orais and STIMs: physiological mechanisms and disease. J. Cell Mol. Med..

[4] Mukherjee S., Brooks W.H. (2014). Stromal interaction molecules as important therapeutic targets in diseases with dysregulated calcium flux. Biochim. Biophys. Acta.

[5] Johnstone L.S., Graham S.J., Dziadek M.A. (2010). STIM proteins: integrators of signalling pathways in development, differentiation and disease. J. Cell Mol. Med..

[6] Zhang W., Trebak M. (2011). STIM1 and Orai1: novel targets for vascular diseases?. Sci. China Life Sci..

[7] Vashisht A., Trebak M., Motiani R.K. (2015). STIM and Orai proteins as novel targets for cancer therapy. A review in the theme: cell and molecular processes in cancer metastasis. Am. J. Physiol. Cell Physiol..

[8] Tanwar J., Trebak M., Motiani R.K. (2017). Cardiovascular and hemostatic disorders: role of STIM and Orai proteins in vascular disorders. Adv. Exp. Med. Biol..

[9] Motiani R.K., Tanwar J., Raja D.A., Vashisht A., Khanna S., Sharma S. (2018). STIM1 activation of adenylyl cyclase 6 connects Ca(2+) and cAMP signaling during melanogenesis. EMBO J..

[10] Lacruz R.S., Feske S. (2015). Diseases caused by mutations in ORAI1 and STIM1. Ann. N. Y Acad. Sci..

[11] Tedeschi V., La Russa D., Franco C., Vinciguerra A., Amantea D., Secondo A. (2021). Plasma membrane and organellar targets of STIM1 for intracellular calcium handling in health and neurodegenerative diseases. Cells.

[12] Johnson M.T., Gudlur A., Zhang X., Xin P., Emrich S.M., Yoast R.E. (2020). L-type Ca(2+) channel blockers promote vascular remodeling through activation of STIM proteins. Proc. Natl. Acad. Sci. U. S. A..

[13] Johnson M.T., Xin P., Benson J.C., Pathak T., Walter V., Emrich S.M. (2022). STIM1 is a core trigger of airway smooth muscle remodeling and hyperresponsiveness in asthma. Proc. Natl. Acad. Sci. U. S. A..

[14] Jardin I., Rosado J.A. (2016). STIM and calcium channel complexes in cancer. Biochim. Biophys. Acta.

[15] Brenner M., Hearing V.J. (2008). The protective role of melanin against UV damage in human skin. Photochem. Photobiol..

[16] Raposo G., Marks M.S. (2007). Melanosomes--dark organelles enlighten endosomal membrane transport. Nat. Rev. Mol. Cell Biol..

[17] Marks M.S., Heijnen H.F., Raposo G. (2013). Lysosome-related organelles: unusual compartments become mainstream. Curr. Opin. Cell Biol..

[18] Natarajan V.T., Ganju P., Ramkumar A., Grover R., Gokhale R.S. (2014). Multifaceted pathways protect human skin from UV radiation. Nat. Chem. Biol..

[19] Videira I.F., Moura D.F., Magina S. (2013). Mechanisms regulating melanogenesis. Bras Dermatol..

[20] Abdel-Malek Z.A., Swope V.B., Starner R.J., Koikov L., Cassidy P., Leachman S. (2014). Melanocortins and the melanocortin 1 receptor, moving translationally towards melanoma prevention. Arch. Biochem. Biophys..

[21] Levy C., Khaled M., Fisher D.E. (2006). Mitf: master regulator of melanocyte development and melanoma oncogene. Trends Mol. Med..

[22] Costin G.E., Hearing V.J. (2007). Human skin pigmentation: melanocytes modulate skin color in response to stress. FASEB J..

[23] Goding C.R. (2000). Mitf from neural crest to melanoma: signal transduction and transcription in the melanocyte lineage. Genes Dev..

[24] Tanwar J., Saurav S., Basu R., Singh J.B., Priya A., Dutta M. (2022). Mitofusin-2 negatively regulates melanogenesis by modulating mitochondrial ROS generation. Cells.

[25] Sultan F., Basu R., Murthy D., Kochar M., Attri K.S., Aggarwal A. (2022). Temporal analysis of melanogenesis identifies fatty acid metabolism as key skin pigment regulator. PLoS Biol..

[26] Im S., Moro O., Peng F., Medrano E.E., Cornelius J., Babcock G. (1998). Activation of the cyclic AMP pathway by alpha-melanotropin mediates the response of human melanocytes to ultraviolet B radiation. Cancer Res..

[27] Arora S., Tanwar J., Sharma N., Saurav S., Motiani R.K. (2021). Orai3 regulates pancreatic cancer metastasis by encoding a functional store operated calcium entry channel. Cancers (Basel).

[28] Walton K.M., Rehfuss R.P., Chrivia J.C., Lochner J.E., Goodman R.H. (1992). A dominant repressor of cyclic adenosine 3',5'-monophosphate (cAMP)-regulated enhancer-binding protein activity inhibits the cAMP-mediated induction of the somatostatin promoter *in vivo*. Mol. Endocrinol..

[29] Barco A., Alarcon J.M., Kandel E.R. (2002). Expression of constitutively active CREB protein facilitates the late phase of long-term potentiation by enhancing synaptic capture. Cell.

[30] Shibahara S., Takeda K., Yasumoto K., Udono T., Watanabe K., Saito H. (2001). Microphthalmia-associated transcription factor (MITF): multiplicity in structure, function, and regulation. J. Investig. Dermatol. Symp. Proc..

[31] Zambelli F., Pesole G., Pavesi G. (2009). Pscan: finding over-represented transcription factor binding site motifs in sequences from co-regulated or co-expressed genes. Nucl. Acids Res..

[32] Kreft L., Soete A., Hulpiau P., Botzki A., Saeys Y., De Bleser P. (2017). ConTra v3: a tool to identify transcription factor binding sites across species, update 2017. Nucl. Acids Res..

[33] Tang Z., Kang B., Li C., Chen T., Zhang Z. (2019). GEPIA2: an enhanced web server for large-scale expression profiling and interactive analysis. Nucl. Acids Res..

[34] Murre C., McCaw P.S., Baltimore D. (1989). A new DNA binding and dimerization motif in immunoglobulin enhancer binding, daughterless, MyoD, and myc proteins. Cell.

[35] Yu F., Lu Y., Zhong Z., Qu B., Wang M., Yu X. (2021). Mitf involved in innate immunity by activating tyrosinase-mediated melanin synthesis in pteria penguin. Front. Immunol..

[36] Goding C.R., Arnheiter H. (2019). MITF-the first 25 years. Genes Dev..

[37] Lowings P., Yavuzer U., Goding C.R. (1992). Positive and negative elements regulate a melanocyte-specific promoter. Mol. Cell Biol..

[38] Bentley N.J., Eisen T., Goding C.R. (1994). Melanocyte-specific expression of the human tyrosinase promoter: activation by the microphthalmia gene product and role of the initiator. Mol. Cell Biol..

[39] Yavuzer U., Goding C.R. (1994). Melanocyte-specific gene expression: Role of repression and identification of a melanocyte-specific factor, MSF. Mol. Cell Biol..

[40] Aksan I., Goding C.R. (1998). Targeting the microphthalmia basic helix-loop-helix-leucine zipper transcription factor to a subset of E-box elements *in vitro* and *in vivo*. Mol. Cell Biol..

[41] Sallinger M., Tiffner A., Schmidt T., Bonhenry D., Waldherr L., Frischauf I. (2020). Luminal STIM1 mutants that cause tubular aggregate myopathy promote autophagic processes. Int. J. Mol. Sci..

[42] Kawakami A., Fisher D.E. (2017). The master role of microphthalmia-associated transcription factor in melanocyte and melanoma biology. Lab. Invest..

[43] Yardman-Frank J.M., Fisher D.E. (2021). Skin pigmentation and its control: from ultraviolet radiation to stem cells. Exp. Dermatol..

[44] Natarajan V.T., Ganju P., Singh A., Vijayan V., Kirty K., Yadav S. (2014). IFN-gamma signaling maintains skin pigmentation homeostasis through regulation of melanosome maturation. Proc. Natl. Acad. Sci. U. S. A..

[45] Vashisht A., Tanwar J., Motiani R.K. (2018). Regulation of proto-oncogene Orai3 by miR18a/b and miR34a. Cell Calcium.

[46] Giaimo B.D., Ferrante F., Borggrefe T. (2017). Chromatin immunoprecipitation (ChIP) in mouse T-cell lines. J. Vis. Exp..

