# Supplementary figures and images for: MITF is a novel transcriptional regulator of the calcium sensor STIM1: Significance in physiological melanogenesis
Source: J Biol Chem. 2022 Nov 7;298(12):102681. doi: 10.1016/j.jbc.2022.102681 (PMC9723939; doi:10.1016/j.jbc.2022.102681)

# Supplementary Figure 1. MITF silencing decreases STIM1 expression and activity

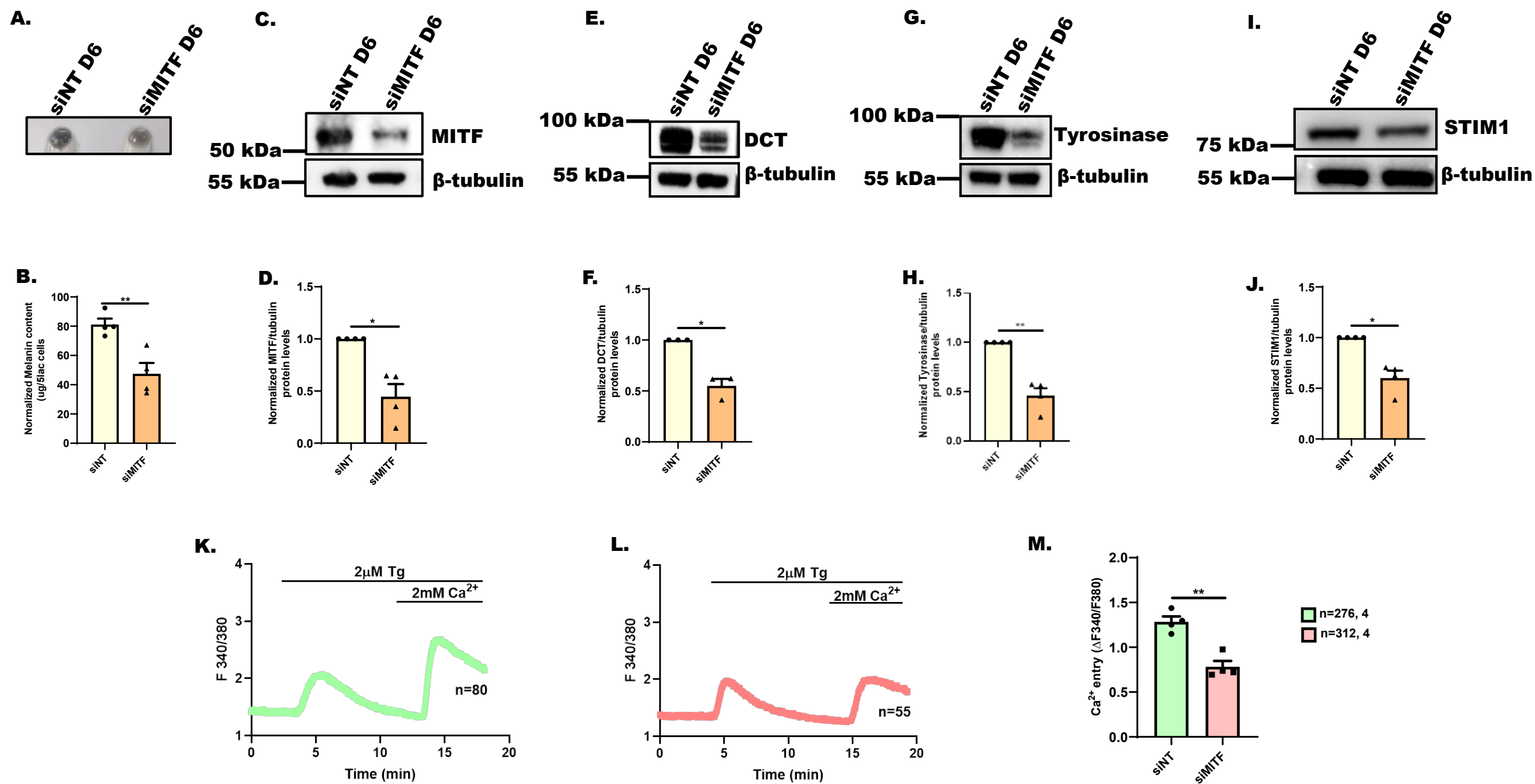

Supplement: Supplemental figure 1 [file mmc1.pdf]
